# Patients With Psychotic Disorders Are More Likely to Refuse Vaccination: An Audit of Vaccine Acceptability on Acute Adult Psychiatric Wards

**DOI:** 10.1192/bjo.2022.467

**Published:** 2022-06-20

**Authors:** Rupert Miles-Marsh, Elias Diamantis, George Duan, Lori Edwards Suarez, Sudheer Lankappa

**Affiliations:** Nottinghamshire Healthcare NHS Trust, Nottingham, United Kingdom

## Abstract

**Aims:**

This audit is looking at COVID-19 vaccine uptake in an acute adult psychiatric setting as part of the national drive to minimize COVID-19 infection. The aims of this audit are to identify: the number of patients that have been offered vaccination in a ward setting; the acceptability of the vaccination and the reasons for non-acceptance of vaccine.

**Methods:**

A total of 339 patients were admitted to acute adult psychiatric wards (Male, Female, PICU) at Highbury Hospital, Nottingham between February to August 2021. Data on the following parameters: demographics (age, sex, ethnicity), section status, HoNOS cluster, admission length and vaccine data (offered, accepted, received) using the RIO system and Health Informatics.

**Results:**

Out of 339 patients, 31% (n = 105) had received or planned to receive the first dose of vaccine prior to admission. 43% (n = 100) of 234 patients who hadn't received vaccine were offered. Out of the patients who were offered vaccine, 59% (n = 59) accepted. 92% (n = 55) of patients who accepted vaccine, received vaccine. Those offered vaccination had an average length of stay of 117 days whilst those not offered had a shorter average length of stay of 81 days.

For patients who were offered vaccine, those who were sectioned and in psychotic clusters refused vaccine compared to non-psychotic and informal patients. Deprivation, gender, age, admission length had no statistical significance in vaccine uptake for patients who were offered.

Patients listed the following reasons for refusing the vaccine: media distrust; vaccine not effective; already had COVID-19; doesn't want it; believes vaccine made by consultant; doesn't want bad reaction; “Scientists and politicians are liars”; “I am fine and don't need it"; “Don't trust it and don't like needles”; “Don't want to be part of the game”; “Have had covid twice and, if I get it, I'd prefer my body to fight it”.

**Conclusion:**

Our current vaccine acceptance rate of 59% is lower than those found nationally (80%) and in a medium secure psychiatric hospital (77%). The trust policy recommends all eligible patients should be offered the vaccine; our offer rate is lower than this standard. The low offer rate may be explained by vaccines being offered in rounds leading to patients possibly being missed. Our acceptance rate could be enhanced by improving our vaccination care plans for formally admitted psychotic patients.

